# LibGENiE – A bioinformatic pipeline for the design of information-enriched enzyme libraries

**DOI:** 10.1016/j.csbj.2023.09.013

**Published:** 2023-09-14

**Authors:** David Patsch, Michael Eichenberger, Moritz Voss, Uwe T. Bornscheuer, Rebecca M. Buller

**Affiliations:** a Zurich University of Applied Sciences, School of Life Sciences and Facility Management, Institute of Chemistry and Biotechnology, Einsiedlerstrasse 31, 8820 Wädenswil, Switzerland; b Institute of Biochemistry, Department of Biotechnology & Enzyme Catalysis, Greifswald University, Felix-Hausdorff-Strasse 4, 17487 Greifswald, Germany

**Keywords:** Bioinformatic tools, Enzyme engineering, Library design, Sequence space

## Abstract

Enzymes are potent catalysts with high specificity and selectivity. To leverage nature’s synthetic potential for industrial applications, various protein engineering techniques have emerged which allow to tailor the catalytic, biophysical, and molecular recognition properties of enzymes. However, the many possible ways a protein can be altered forces researchers to carefully balance between the exhaustiveness of an enzyme screening campaign and the required resources. Consequently, the optimal engineering strategy is often defined on a case-by-case basis. Strikingly, while predicting mutations that lead to an improved target function is challenging, here we show that the prediction and exclusion of deleterious mutations is a much more straightforward task as analyzed for an engineered carbonic acid anhydrase, a transaminase, a squalene-hopene cyclase and a Kemp eliminase. Combining such a pre-selection of allowed residues with advanced gene synthesis methods opens a path toward an efficient and generalizable library construction approach for protein engineering. To give researchers easy access to this methodology, we provide the website LibGENiE containing the bioinformatic tools for the library design workflow.

## Graphical Abstract

**Figure:**
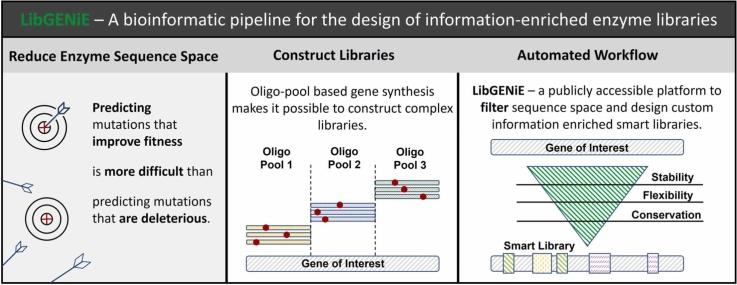

## 1 Introduction

**Fig. 1:**
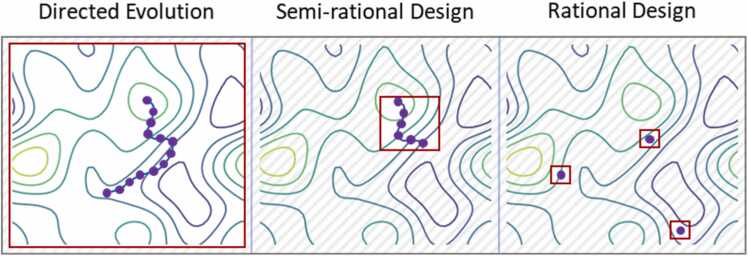
Overview of protein engineering techniques. The different categories are sorted by their required screening effort from left (highest) to right (lowest). In traditional directed evolution, the sequence space (red box) is commonly explored randomly, with little additional information required. Rational design can be viewed as a complementary approach. Information about the system, which can include experimental data, knowledge of the mechanism, as well as computational techniques, is used to reduce the sequence space as much as possible, and areas within it are sampled selectively. Semi-rational design also relies on additional information to reduce the screening space; however, experiments and physical evaluation are still required. Notably, the boundaries between these techniques are often fluid, and the optimal engineering method depends on many factors, such as the complexity of the functional assay, available screening capabilities, or previous knowledge of the enzyme. Image inspired by Bornscheuer et al. [14].

Enzymes are remarkable catalysts capable of facilitating complex reactions with high substrate specificity and exquisite chemo-, regio- and enantioselectivity [1], [2]. However, when used in conditions necessary to drive a process at an industrial scale, the performance of wild-type enzymes often remains insufficient from an economic standpoint. Thus, to better harness the capabilities of nature's catalysts in industrial settings, much focus has been placed on advancing protein engineering strategies to proficiently tailor enzymes' catalytic, biophysical, and molecular recognition properties [3], [4]. In this way, enzyme engineering has allowed to broaden the substrate scope of natural enzymes [5], change their chemistry [6], improve catalytic activity [7], [8], [9], or alter enantioselectivity [10], [11]. Yet, despite their successful outcome, these protein engineering examples did not explore all possible amino acid configurations of the target enzymes, and consequently, the solutions found in evolution campaigns might be far from optimal. However, since the number of possible enzyme variants scales exponentially with protein sequence length, the screening burden imposed on researchers quickly becomes intractable when attempting to explore enzyme composition comprehensively. For illustration, a protein composed of only 100 amino acids can be altered in 20^100^ ways, an astronomical number far exceeding even the estimated number of atoms in the universe [12]. Faced with this challenge, also called "the numbers problem in directed evolution" [13], protein engineers aim to navigate sequence space as efficiently as possible and constantly seek to develop novel methods to optimize the process. Existing approaches can broadly be classified into the categories of 1) directed evolution, 2) semi-rational, and 3) rational protein design (Fig. 1) and are often employed in accordance with the available screening capabilities and prior information about the enzymatic system [14].

Traditional directed evolution, which relies on gene recombination or whole-gene error-prone PCR to create diversity, is often associated with a heavy screening burden as many of the introduced mutations in the libraries are either neutral or unfavorable [15]. Positively, however, directed evolution does not require any prior knowledge about enzyme function or structure to be effective. In contrast, rational enzyme design [16] aims to limit enzymatic screening efforts to only a few distinct amino acid substitutions [17]. The approach relies on an intimate knowledge of a protein's function and/or structure and, as such, requires high predictive accuracy, which can be obtained – at least in part – through the interpretation of experimental data. Although bioinformatic tools such as AlphaFold 2 [18] have facilitated the access to high quality protein models, rational modulation of crucial residues often requires far more fine-grained information on receptor-ligand interaction networks and dynamics. Additionally, significant *in-silico* efforts might be required to resolve uncertainty around specific mechanisms and illuminate required factors between interaction partners to drive a desired reaction [19]. Even with the advanced bioinformatic methods available today, it can be challenging to rationalize which sites, specific residues, or combinations should be selected when optimizing a protein for a certain task.

Lastly, semi-rational protein engineering fuses elements of rational design and directed evolution to create more focused enzyme libraries of higher quality [4], [20]. This combination leads to a more efficient sampling of the sequence space, resulting in a lower screening burden than completely random approaches [21], [22] while allowing more leniency for computational limitations and inaccuracies. For example, researchers can investigate the 3D structure of an enzyme to identify the catalytic pocket and focus their engineering efforts only on this region which is likely to react more directly to amino acid exchanges. In this way, sequence space can be reduced while beneficial mutations can be largely sampled, as many of them are typically situated in the active site [23], [24]. In practice, researchers often aggregate information from sources such as the target enzyme's 3D structure, function, previous knowledge (for example, mutational data), phylogeny, docking, or machine learning to preselect potential hotspots [16], [20]. Based on this information, focused libraries ranging in size from ∼200–2000 enzyme variants are constructed. Such screening efforts are within the scope of what GC or HPLC systems can handle within a reasonable timeframe [25]. It should be noted, though, that semi-rational enzyme design also suffers from the "numbers problem in directed evolution", and in many cases, only a small fraction of the targeted variants can be analyzed experimentally. In addition, experimental throughput is hampered by limitations in the physical construction of complex gene libraries.

Using standard molecular biology strategies, the creation of large, randomized libraries through methods such as error-prone PCR or the construction of a few specific variants through site-directed mutagenesis is easily possible. However, building large libraries made up of predefined enzyme variants often remains expensive and challenging. One exciting prospect to address the existing library construction bottlenecks is the use of micro-array-based "oligo-pools". These pools are mixes of up to several hundred thousand individually designed polynucleotides with < 300 bp length, synthesized through phosphoramidite chemistry [26]. Notably, array-based oligo synthesis is orders of magnitude cheaper than traditional column-based synthesis routes, with costs ranging from US$ 0.00001–0.001 per nucleotide, depending on length, scale, platform, or vendor [27]. Considering a typical library size for semi-rational enzyme design (< 2000 variants) and a protein of approximately 300 amino acid length, oligo pools for focused libraries can consequently be ordered for roughly 2000 US$ [28], leading to material costs of approximately 1 US$ per variant. Consequently, despite issues like truncated DNA molecules and high error rates [29], the oligo-pool option could be more cost-effective than degenerate or reduced codon coverage primers traditionally employed for library construction strategies while allowing for much more flexibility in library design.

Relevant enzymatic properties to be optimized for industrial applications include activity, thermo- and solvent stability, selectivity, and specificity [30]. As delineated above, reliably selecting appropriate amino acid residues for randomization to improve any of these traits is a challenging aspect of semi-rational enzyme design. Guiding principles might be to select residues near the binding pocket to engineer enantioselectivity [11] or substitute specific residues to redesign unstable protein regions to improve thermostability [31]. Especially the latter, namely the modulation of protein stability through the introduction of mutations, is a widely pursued goal, and different computational procedures have been established to this end, including the use of sophisticated physical force fields, deep learning, and hybrid approaches [32], [33], [34], [35], [36], [37], [38].

Intriguingly, computational techniques can be helpful in ways that might not be immediately obvious. For example, we followed the logic that it seems much easier to predict destabilizing mutations than amino acid changes that stabilize a protein scaffold [38]. We consequently reasoned that methods developed to predict enzyme sequences with improved stability might be used in a much broader sense if they were uniquely used to identify destabilizing mutations. Through the exclusion of such destabilizing mutations, the design of solution-enriched enzyme libraries for the optimization of enzyme activity or any other desirable traits would be made possible. The resulting complex libraries could then, in turn, effectively be built using specifically designed oligo-pools.

The ease of access to a new methodology plays a major role in its adoption [10]. Popular protein engineering tools such as PROSS [38], [39], HotSpot Wizard [40] as well as FuncLib [41] and htFunclib [42] play a significant role in bridging the gap between computational and biological skills, allowing for faster and more efficient evolution campaigns [43]. The enumerated web servers help researchers design more stable enzymes, identify mutational hot spots, or develop specific multiple-point mutants in active sites to improve activity, respectively. Complementing these tools, we introduce LibGENiE, a web platform to pre-filter sequences with the aim to provide researchers with a list of deleterious mutations to exclude from enzyme libraries. Following the filtering step, which can be supplemented with additional information from other protein engineering webservers, LibGENiE can be used to design oligo-pools to construct the complex libraries. These information-enriched libraries will be particularly helpful in evolution campaigns that can accommodate the throughput of hundreds to thousands of variants per round.

## 2 Results

### 2.1 Predicting (and excluding) destabilizing mutations

To set the basis for our approach, we analyzed available literature data of successful evolution campaigns, including data generated during the optimization of a carbonic anhydrase [8], a transaminase [44], a squalene-hopene cyclase [45] and a Kemp eliminase [7]. In a first step, we calculated the ΔΔG values, a measure of free energy changes upon mutation [34], for all possible amino acid substitutions at all sites in the selected wild-type enzymes using a cartesian ΔΔG protocol implemented in the Rosetta Protein Modelling Suite [46]. For example, in the case of an enzyme consisting of 300 amino acids, all possible 20 * 300 ΔΔG values were calculated. These ΔΔG values can help approximate how mutations affect protein stability by comparing the free energy of the native and altered conformation of a protein. Negative values typically refer to a stabilizing mutation, while strongly positive values denote destabilizing mutations.

**Fig. 2:**
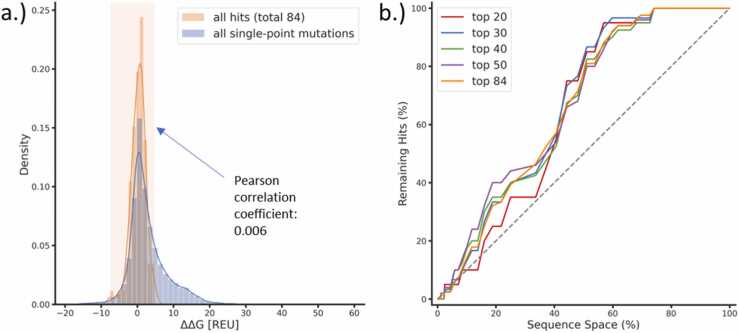
a.) Density plot of predicted ΔΔG values (lower values correspond to higher predicted stability) of a carbonic anhydrase [8]. The blue density curve depicts the ΔΔG values of all possible single-point mutants, and the orange plot represents the ΔΔG distribution of the 84 beneficial single-point variants identified in the first round of carbonic anhydrase evolution. The ΔΔG range in which hits were identified is highlighted in orange. Additionally, the Pearson correlation coefficient between the activity of identified hits and predicted ΔΔG is shown. b.) Line chart of the same dataset as in a.). The x-axis refers to the sequence space when reducing it only through predicted ΔΔG values. For example, if we remove the variants with the highest 10% of predicted ΔΔG values (most destabilizing), 90% of the sequence space remains. The y-axis represents how many of the 84 reported hits can be found in a given remaining sequence space. For example, none of the 84 reported hits are within the sequence space characterized by the highest 10% predicted ΔΔG values. This analysis is shown for the 20, 30, 40, 50, and 84 best-measured hits (out of 84). As a comparison, the gray dashed line highlights the impact of reducing the sequence space randomly.

Following this protein-wide stability profiling, we analyzed in which range the ΔΔG values of the experimentally determined beneficial mutations of the selected enzymes were located: For example, we studied data generated by Codexis, a US-based company specialized in protein engineering, which evolved a carbonic anhydrase towards improved activity at higher temperatures. To do so, the researchers saturated all non-catalytic residues in a first evolution round [8], identifying 84 unique carbonic anhydrase variants that performed better than the wild-type under their screening conditions. Our ΔΔG analysis indicated that most of the mutations observed in improved variants were within the lowest (stabilizing) 60% of predicted ΔΔG values hinting that a large part of the screening space could have been excluded a priori (Fig. 2b). Interestingly, we noted that while we could identify destabilizing mutations, the predicted ΔΔG values became much less informative beyond a certain exclusion threshold. In general, it is estimated that 0.01 – 1% of all mutations are beneficial [47]. In the ΔΔG range where most of these improved enzyme variants were found (−7.5 to 4.7 Rosetta energy units (REU), Fig. 2a), the measured fold improvement over wild-type did not show a correlation to the calculated ΔΔG values (Pearson correlation coefficient 0.006, Fig. 2a).

To test the general applicability of this finding with examples from distinct enzyme families beyond enzyme class 4 (carbonic anhydrase), we turned to analyze the evolutionary trajectories of enzymes stemming from enzyme class 2 (transaminase), enzyme class 5 (squalene hopene cyclase) as well as a computationally designed enzyme (Kemp eliminase) based on a scaffold from enzyme class 3 (xylanase). The transaminase ATA-217, engineered towards synthesizing a chiral precursor of sacubitril, an active ingredient in the blockbuster drug Entresto, harbored 26 mutations in the final variant [44] whereas four mutations allowed the squalene hopene cyclase *Aci*SHC to gain enantio-complementary access to valuable monocyclic terpenoids [45]. Kemp eliminase HG3, a computationally designed enzyme capable of catalyzing a proton abstraction reaction from 5-nitrobenzisoxazole, was optimized in 17 rounds of directed evolution to yield a variant with 17 mutations whose catalytic activity rivals that of natural enzymes (*k*_cat_ = 700 ± 60 s^−1^, *k*_cat_/*K*_m_ = 230,000 ± 20′000 s^−1^ M^−1^) [7].

In all investigated evolution projects, we observed the general trend that destabilizing mutations were not incorporated in evolved enzyme variants. Notably, when comparing amino acid mutations predicted to be destabilizing as single point mutations in the wild-type enzymes to any reported beneficial single point mutation within the evolution campaigns (Fig. S1/S2), we deduced that almost all the strongly destabilizing mutations could be excluded confidently at the outset of the enzyme optimization projects (Table 1, Table S1, Fig. S1, Fig. S2). Concretely, from our datasets, we observe that 30–50% of mutations have a strongly destabilizing effect, which is in good agreement with previous reports [47], [48], [49], [50], [51], [52]. This sequence space can be thus cut confidently from the outset of library design. Interestingly, in the case of evolved *Aci*SHC, we observed a single outlier: Mutation A169P was flagged as destabilizing (21.5 REU) yet still appeared in the optimized squalene-hopene cyclase variant. Potentially, the destabilizing mutation was incorporated because *Aci*SHC is a thermophilic enzyme whose scaffold would generally allow for more leeway toward introducing destabilizing mutations.

**Table 1:** Overview of how ΔΔG values of single mutations found in the final improved variants of the selected evolution campaigns are distributed within the context of all possible calculated ΔΔG values for the wild-type enzymes. In this analysis, the most destabilizing mutations in the context of the wild-type enzyme are gradually removed (in 10% steps), reducing the theoretical sequence space from left to right. The remaining sequence space is analyzed with respect to its harboring the amino acid substitutions found in evolved enzyme variants and the value is given in percent (%). For example, in the case of HG3 evolution, a focused library in which the 40% most destabilizing mutations are removed from sequence space would still contain all the 17 beneficial mutations identified in the final variant.

		**Sequence space (%)**											
**Dataset**			**# Mut**	**100**	**90**	**80**	**70**	**60**	**50**	**40**	**30**	**20**	**10**
	**ATA217**		26	100	100	96.2	92.3	88.5	73.1	73.1	61.5	53.8	42.3
	**HG3.17**		17	100	100	100	100	100	82.4	52.9	52.9	47.1	41.2
	**DvCA**		36	100	100	100	97.2	91.7	77.8	61.1	44.4	38.9	13.9
	***Aci*****SHC**		4	100	75	75	75	75	75	75	50	50	50
											
	**average**			100	93.8	92.8	91.1	88.8	77.1	65.5	52.2	47.4	36.8

Conclusively, the relationship between activity and stability is often complex, with reports of both negative [53], [54], [55], [56] and positive correlations [57], [58] between stability and function attesting to the fact that different enzymatic systems behave differently to mutations. Strikingly, as highlighted in this work, employing the opposite approach for the construction of information-enriched libraries seems much more reliable: Strongly destabilizing mutations are often accompanied by a loss in function (Table 1, Fig. 2), consequently enabling their early exclusion from the sequence pool.

### 2.2 Oligo pools for library creation

Promisingly, as seen above, reducing the amino acid alphabet in gene library preparation can be facilitated through computational techniques. Yet, it is equally important to have in mind that such a process might lead to libraries that are too diversified to be easily and economically constructed. In this respect, it is important to consider the redundant nature of the genetic code in which the 20 natural amino acids are encoded by 61 sense codons. In consequence, researchers have tried to avoid using the heavily redundant NNN codon in library construction which additionally suffers from the occurrence of stop codons (N standing for any of the four DNA bases). Instead, they have turned to using primers harboring degenerate codons such as NNK (32 codons, 20 amino acids), NDT (12 codons, 12 amino acids) or using the 22c (22 codons, 20 amino acids), and 20c (20 codons, 20 amino acids) tricks [13], [59], [60].

**Fig. 3:**
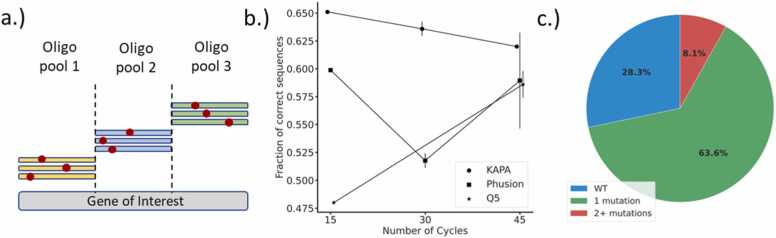
a.) As oligonucleotides ordered within oligo-pools are limited to < 300 bp in length, the target gene must be split into smaller fragments below this size. These mutations can then be introduced into the desired gene through standard molecular biology techniques such as SOEing [61]. b.) Fraction of correct sequences in the amplified oligo-pool. The experiments were conducted with varying amounts of PCR cycles (15, 30 and 45), as well as different polymerases (Q5, Phusion, KAPA). The error bars denote the average and error of experiments that vary in their dNTP concentration. c.) Overview of library quality resulting from fragment amplification with KAPA polymerase using 30 amplification cycles. Sequencing highlighted that 63.6% of variants were produced correctly (one desired mutation – green), while 28.3% wildtype sequences (blue) and 8.1% sequences that contain two or more mutations were observed (red).

Unfortunately, the current strategies using degenerate codons are not suitable to build the information-enriched libraries stemming from our computational workflow, in which each targeted mutation site would demand the inclusion of only certain amino acids (Fig. S3). Thus, we set out to evaluate the feasibility of using micro-array-synthesized oligonucleotides, commercially available under the term "oligo-pools", for constructing the complex libraries derived from our stability filtering strategy (Fig. 3a, Fig. S3).

In particular, we opted to focus our attention on single-point residue exchanges. As there are limitations to the synthesis length of oligo-pools [29], desired mutations must be split across multiple fragments or "sub-pools" (Fig. 3a), which can be separated from the main pool with sub-pool specific primers. These sub-pools consist of individual oligonucleotides, each carrying a single mutation, which can be introduced into the gene of interest through traditional molecular biology techniques, such as gene splicing by overlap extension PCR (SOEing) [61].

To evaluate the suitability of the oligo-pools for the construction of tailored enzyme variant libraries, we ordered a pool of 200 oligo sequences encoding the initial 157 bases of the Kemp eliminase HG3 [7]. To create diversity for sequence analysis, three consecutive adenine nucleotides were introduced within four spatially distinct regions of the 157 bp gene fragment (sequence A: bp 30 – 32; sequence B: bp 62 – 64; sequence C: bp 93 – 94; sequence D: bp 124–126) and each such sequence was ordered in the pool fifty times. Following fragment amplification and cloning, we noted relatively high rates (∼50%) of undesirable sequences, split between either wild-type sequences or multiple-point mutants (Fig. 3b). This high fraction of incorrect sequences was not wholly unexpected and correlates to the range reported in previous projects that leverage oligo pools for single-point mutation library creation [62], [63], [64].

Oligo pools suffer from the low concentration of individual oligonucleotides [29] making an initial amplification step indispensable [65]. In fact, depending on the number of projects combined within one oligo pool, it might be required to perform this amplification twice: once to isolate the sub-pools [66] and then again to separate the individual fragments. We suspected that these PCR amplification steps introduce additional errors into the oligo-pool libraries through uncoupling events that lead to truncated PCR products. These truncated gene products can serve as primers during the next PCR cycle [67], [68], either picking-up additional mutations (leading to multiple-point variants) or overwriting desired mutations altogether (resulting in wild-type). As the prevalence of PCR abortions is affected by multiple factors, such as the concentration of nucleotides, the number of PCR cycles, and the polymerase used for amplification [69], we opted to optimize the amplification procedure.

To do so, we investigated ways how to improve the overall sampling efficiency of oligo-libraries by testing different polymerases (Q5, Phusion, and KAPA polymerase), dNTP concentrations, and varying amounts of PCR cycles (15, 30, and 45) for their impact on the formation of undesired gene fragments. Using the same oligo-pool analysis set-up as described previously, it became clear that neither the dNTP concentration nor the number of PCR cycles significantly impacted the number of corrupted sequences (Fig. S4). However, the choice of polymerase showed an influence on gene fragment integrity (Fig. 3b): While Q5 and Phusion polymerase led to 47.5 – 60% correct fractions, KAPA polymerase was found to be most suited for oligo-pool amplification (> 60% correct fragments). The remaining undesirable sequences were split between wild-type (28.3%) and primarily double-point mutants (8.1%) (Fig. 3c). In summary, we advise that these rates should be considered when designing the sampling strategy of directed evolution studies.

## 3 LibGENiE: a webserver for smart library creation

To facilitate the design of solution-enriched gene libraries and their subsequent construction with the oligo-pool technique, we set up a web server named LibGENiE (available at www.libgenie.ch).

LibGENiE provides data sets compiling common protein properties relied upon in rational design, including phylogenetic conservation (extracted from a multi-sequence alignment generated from three rounds of PSI-BLAST with default settings [70]), stability (predicted from protein free energy changes upon point mutations, ACDC-NN [71]), and flexibility (generated from MEDUSA [72]).

These tools were chosen based on open access (e.g., license situation) and computational demands. For example, Rosetta, a highly accurate and widely used modeling tool (Table S1), can only be freely accessed by academic users and government laboratories. Additionally, the computational resources required to perform stability predictions with Rosetta for all possible single-point mutations in a target protein can be prohibitive for a free-of-charge webserver. With these limitations in mind, we designed the webserver LibGENiE with the intention of giving the broadest possible access to the filtering and oligo design methodology. Complementary information, such as ΔΔG calculations by other methods (for a comparison of available methods please consult [73]), knowledge about relevant amino acid sites, the location of the active pocket, tunnels, or hot spots derived from alternative predictive tools (e.g., HotSpot Wizard [40], Caver [74], PLIP [75], or FireProsASR [76]) can be valuably employed to further fine-tune the filtering step.

**Fig. 4:**
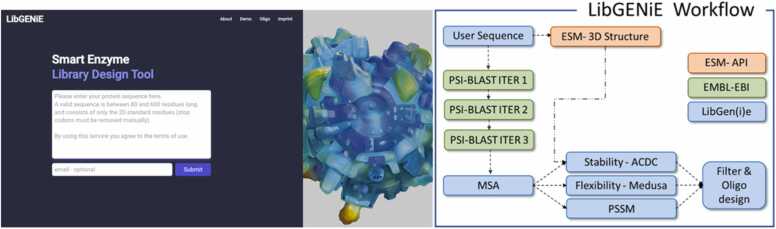
Schematic overview of the LibGENiE landing page and workflow. Based on the user input sequence, three rounds of PSI-BLAST are performed through the EMBL-EBI API [70]. The acquired multiple sequence alignment (MSA) information is then further processed to predict stability (ACDC-NN [71]), flexibility (MEDUSA ([72]), and conservation (MSA from PSI-BLAST). LibGENiE provides raw access to this data, which can be used to restrict the sequence space. In addition, LibGENiE offers a tool for the design of oligo sequences.

Following library design, LibGENiE allows to generate custom oligonucleotides for library construction (Fig. 4), which can be designed based on the preceding *in-silico* filtering. In addition, based on a selected maximum gene length, LibGENiE splits the input sequence into even sections and designs the required amplification primers.

Initializing LibGENiE only requires the user to provide a protein sequence in the range of 80 – 600 residues. From this, a sequence alignment for the input sequence is generated through three rounds of iterative PSI-BLAST [70]. As detailed below, the multiple sequence alignment then serves as the foundation for all further processing.

### 3.1 Thermodynamic stability

Quantifying the change in free energy between the wild-type protein and a single point variant is mainly associated with expression or stability optimization; however, as delineated above, knowing which residues completely destabilize an enzyme provides a valuable input to reduce sequence space of enzyme libraries dedicated to the optimization of functions beyond these enzyme characteristics. To allow filtering of sequence space, LibGENiE will initially attempt to predict the stability of each possible single site variant from the corresponding protein sequence employing the structure-based version of ACDC-NN, an antisymmetric neural network [71]. The structure required to run the algorithm is modeled through the ESM-esmfold_v1 API [77]. If no 3D structure of the protein of interest can be modeled, LibGENiE falls back to sequence-only predictions through ACDC-NN Seq, a model that has been described to favorably compare with other state-of-the-art sequence-based prediction tools as well as some structure-based ones [78].

Even though the Rosetta cartesian-ΔΔG protocol outperforms ACDC-NN on our benchmark datasets (Table S1 and S2), it is important to note that inference on ACDC-NN is orders of magnitude faster and published under a very permissive license allowing to give unrestricted access to a broad user community. As delineated above, fine-tuning of the filtering step with information obtained from complementary web servers can be used to further reduce the size of the resulting information-enriched libraries.

### 3.2 Evolutionary information

Using the MSA, the observed conservation percentages of all 20 amino acids at each position is calculated. This information might be used to "restrict" the allowed sequence space or implement consensus/frequency ratio-based engineering techniques. The intuition behind restricting the allowed sequence space – which is to exclude residues that are never observed in closely related wild-type enzymes – is that deleterious mutations tend to be purged by natural selection [38]. Consensus or frequency ratio techniques introduce changes where the wild-type residue diverges the most from the most common amino acid (consensus) in the multiple sequence alignment. Such changes have been observed to increase stability [79], [80], [81], [82], [83], [84] and are explained in detail by Damborsky et al. in their publication accompanying the release of HotSpot Wizard 2.0 [85].

### 3.3 Structural flexibility

Introducing mutations to rigidify flexible positions can yield proteins with improved stability [86]. This technique builds on the notion that selective substitutions of mobile residues can introduce additional interactions/contacts between neighbors [87], [88], causing enhanced rigidity, which in turn leads to higher thermostability [89]. A typical experimental metric for protein flexibility is the B-factor, which reflects the X-ray scattering caused by thermal motion [90]. However, as B-factors are an experimental metric, and crystal structures are not available for all proteins, computational tools have been developed to predict them. In LibGENiE, we provide predictions of flexibility from one such tool, MEDUSA [72], a deep-learning-based protein flexibility model trained on experimentally determined B-factor values.

### 3.4 Oligo design

As outlined above, oligo pools are limited in length. To enable the introduction of single point mutations at any desired position within a target sequence, the gene must consequently be split into smaller sections. Based on the provided input DNA sequence, LibGENiE’s oligo design tool divides the gene into fragments of desired length including all targeted single-point mutations. In addition, the sequences of the required amplification primers are designed.

## 4 Conclusion

Semi-rational protein engineering is an elegant compromise between directed evolution and rational design. It directly addresses the screening bottleneck of classical directed evolution while circumventing the need to have an absolute understanding of the sequence-function relationship in enzymes (and, consequently, the required computational resources). To conduct semi-rational protein engineering, several strategies to reduce sequence space have been developed and allowed the construction of powerful enzymes for synthesis [16], [21], [60], [91]. In this spirit, we present how the prediction and removal of destabilizing mutations in gene libraries is an effective way to reduce sequence space resulting in information-enriched gene libraries for functional screening.

However, when reducing sequence space, practical “wet-lab" experimental considerations also must be taken into account. Arbitrarily complex libraries cannot be constructed economically in most cases. Thus, improved DNA synthesis techniques will be essential to fuel the demands of an age defined by ever-increasing automation and powerful and accessible DNA sequencing instrumentation. In this vein, on-chip solid-phase gene synthesis presents itself as a compelling asset to semi-rational design as it allows to rapidly construct diverse and complex gene libraries [92]. Using this technology, researchers can build libraries tailored to their screening capabilities that can be scaled dynamically, often with no additional molecular biology overhead.

To facilitate the adoption of mutational pre-filtering, for example through the exclusion of destabilizing mutations, we introduce the webserver LibGENiE for the construction of information-enriched gene libraries. By providing data sets comprising selected common metrics used for protein engineering, LibGENiE affords researchers with a starting point for identifying hot spots and a way to restrict the sequence space to match the bounds of their screening capabilities. LibGENiE was designed to be easily extendable with additional information, whether from already available web servers for protein design such as PROSS [38], HotSpot Wizard [40] and 3DM [93] or other computational tools. In fact, unlike other platforms, LibGENiE provides information for all possible single-point mutants in a user's input sequence rather than suggesting preselected variants or hot spots. By providing unprocessed data, users of LibGENiE have more flexibility to introduce additional custom information and to define the number of variants to be evaluated, which can range from hundreds to thousands, depending on screening capabilities.

## 5 Materials and methods

### 5.1 Data

The enzyme engineering datasets used for analysis were obtained from published manuscripts [7], [8], [44], [45]. The dataset of single mutations in ATA217 [44] was generated by extracting the 26 mutations introduced in the final variant compared to the wild-type sequence. The same procedure was applied to obtain the HG3.17 dataset [7]. The 84 beneficial mutations and their activity for the DvCA dataset were published in the supplement information of [8]. The beneficial mutations for *Aci*SHC stem from publication [45]. Beneficial single-site mutations refer to the highlighted beneficial variants obtained from a 14 single-site saturation screen (Table S1).

### 5.2 Cartesian ΔΔG protocol

ΔΔG predictions were based on a protocol published by the official Rosetta forums: https://www.rosettacommons.org/node/11126. Each mutant was predicted three times, and the lowest energy obtained was compared to the wild-type energies to calculate differences in free energy. The protocol has been adapted from the original publication [34].

### 5.3 Oligo design

A pool of 200 oligo sequences with a length of < 200 bp was ordered from Twist Bioscience. The sequence used were the first 157 bases of the Kemp eliminase HG3 [7]:

TGGCAGAAGCAGCACAGAGCGTTGACCAGCTGATTAAAGCACGTGGTAAAGTTTATTTTGGTGTTGCCACCGATCAGAATCGTCTGACCACCGGTAAAAATGCAGCAATTATTCAGGCAGATTTTGGTATGGTTTGGCCTGAAAATAGCATGAAAT.

Four distinct spatial regions along the 157 bp fragments were changed to three consecutive adenines to create diversity for analysis. Each sequence was ordered 50 times in the pool.

SeqA index: 30, 31, 32; SeqB index: 62, 63, 64; SeqC index: 93, 94, 95; SeqD index: 124, 125, 126.

The full sequences are listed in the supplementary information.

### 5.4 Oligo pool amplification

The oligo pools were amplified according to the protocol provided by Twist Bioscience [65]. For optimization purposes, the final dNTP concentrations (0.3 mM each dNTP or 0.6 mM each dNTP), DNA polymerase (KAPA HiFi HotStart DNA Polymerase (Roche KK2601), Q5 High-Fidelity DNA Polymerase (NEB #M0493), and Phusion High-Fidelity DNA Polymerase (NEB #M0530S) and the number of amplification cycles (15, 30, 40) were changed.

### 5.5 Amplified pool sequencing

After PCR amplification, the PCR pools were prepared, sequenced, and analyzed using Nanopore sequencing according to the protocol outlined in [94]. Correct sequences in which the expected nucleotide changes were detected were annotated as “1 mutation” (Fig. 3c). Sequences harboring no or multiple mutations were classified as wild-type or multiple-point variants, respectively.

## CRediT authorship contribution statement

**David Patsch:** Methodology, Data collection, Software implementation, Formal analysis, writing, Conceptualization. **Michael Eichenberger:** Conceptualization, Methodology. **Moritz Voss:** Conceptualization, Writing - original draft. **Uwe Bornscheuer:** Conceptualization, writing, Writing - review & editing. **Rebecca Buller:** Conceptualization, Methodology, writing, Supervision, Funding acquisition, Project administration, Writing - original draft, Writing - review & editing.

## Declaration of Competing Interest

The authors declare the following financial interests/personal relationships which may be considered as potential competing interests. Rebecca Buller reports financial support was provided by Swiss National Science Foundation.
