# Supplementary material for: LibGENiE – A bioinformatic pipeline for the design of information-enriched enzyme libraries
Source: Comput Struct Biotechnol J. 2023 Sep 14;21:4488–96. doi: 10.1016/j.csbj.2023.09.013 (PMC10510078; doi:10.1016/j.csbj.2023.09.013)
Supplement: Supplementary file 1 — Supplementary material. [file mmc1.pdf]

Supporting Information

LibGENiE – A bioinformatic pipeline for the design of information-enriched enzyme libraries

David Patsch[a,b], Michael Eichenberger[a], Moritz Voss[a], Uwe T. Bornscheuer[b] and Rebecca M. Buller[a,*]

[a] Zurich University of Applied Sciences, School of Life Sciences and Facility Management, Institute of Chemistry and Biotechnology, Einsiedlerstrasse 31, 8820 Wädenswil, Switzerland

[b] Institute of Biochemistry, Department of Biotechnology & Enzyme Catalysis, Greifswald University, Felix-Hausdorff-Strasse 4, 17487 Greifswald, Germany

*Corresponding author: Rebecca M. Buller (rebecca.buller@zhaw.ch)

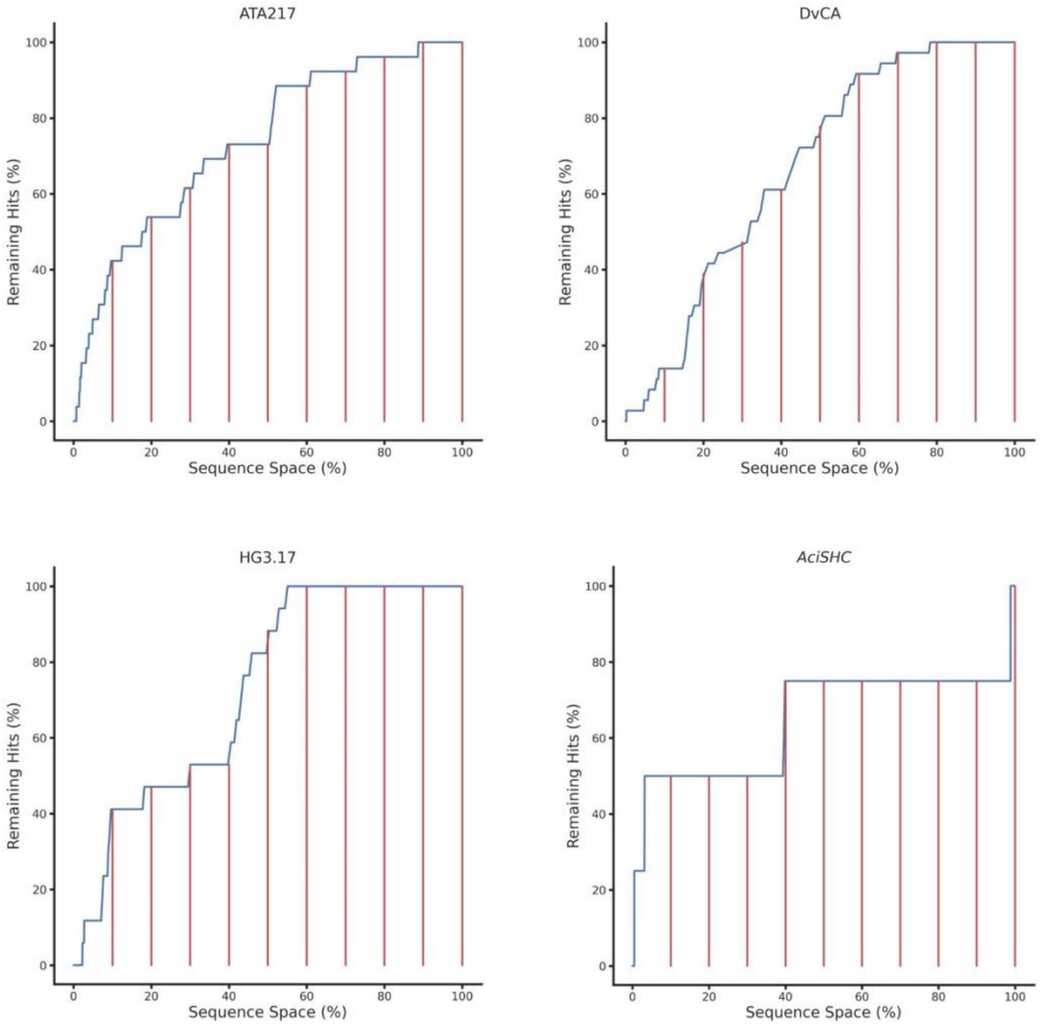

Figure S1: Visual representation of how reductions in sequence space (filtered by ddG) would affect the number of hits identified in the evolutionary trajectories of a transaminase (ATA217) [1], a carbonic anhydrase (DvCA) [2], a computationally designed Kemp Eliminase (HG3.17) [3] and a squalene-hopene cyclase (*Aci*SHC) [4]. The underlying data can be found in Table 1. Vertical red lines are drawn in 10 % increments.

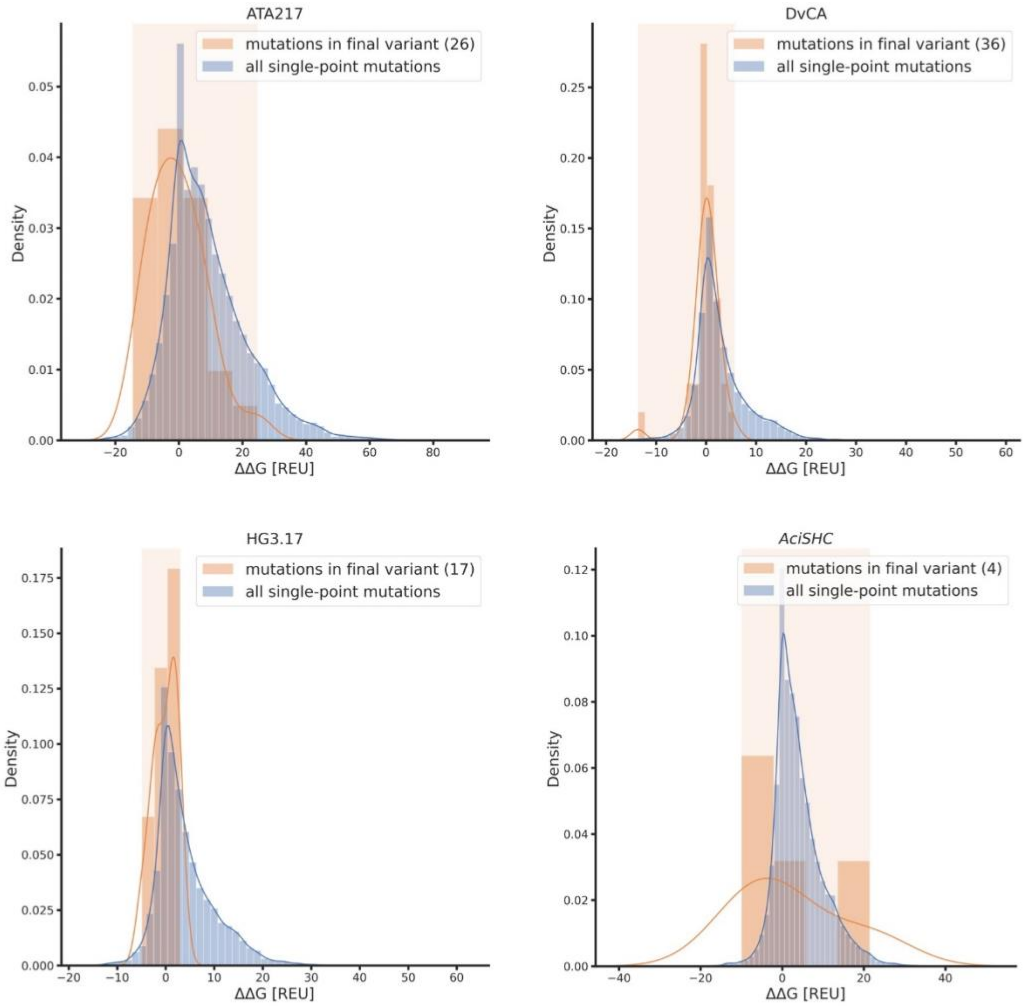

Figure S2: Density plot of predicted ddG values (lower values correspond to higher predicted stability) of transaminase (ATA217) [1], a carbonic anhydrase [2], a computationally designed Kemp Eliminase (HG3.17) [3] and a squalene-hopene cyclase (*Aci*SHC) [4]. The blue density curve depicts the ddG values of all possible single-point mutants, and the orange plot represents the ddG distribution of single-points variants carrying the reported mutations of the final engineered variants individually. The ddG range in which hits were identified is highlighted in orange.

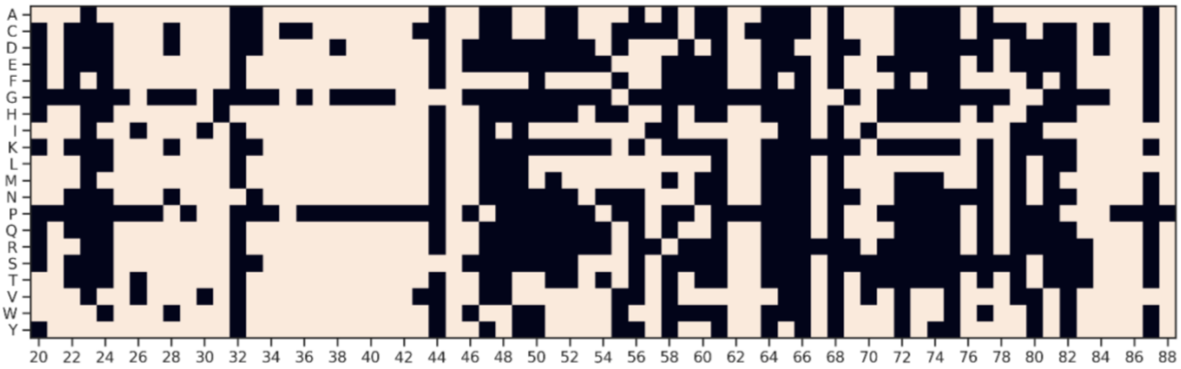

Figure S3: Amino acid distribution of the DvCA gene after removing the 40 % mutants with the highest predicted ddG. Removed residues are colored in black.

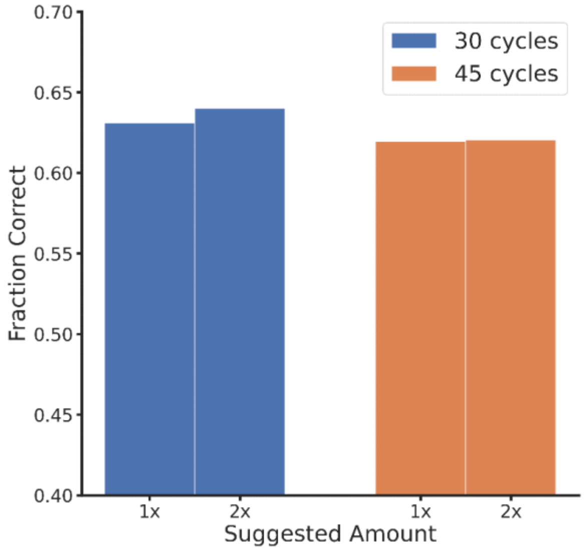

Figure S4: Impact of dNTP concentration on the number of correct sequences for experiments conducted with KAPA polymerase at 30 and 45 cycles. The suggested amount refers to 0.3 mM of each dNTP in the amplification reaction according to the PCR AMPLIFICATION PROTOCOL provided by Twist.

Sequence space (%)

	# mut	100	90	80	70	60	50	40	30	20	10
**ATA217**	**26**	100.0	100.0	96.2	92.3	88.5	73.1	73.1	61.5	53.8	42.3
**HG3.17**	**17**	100.0	100.0	100.0	100.0	100.0	82.4	52.9	52.9	47.1	41.2
**DvCA_Final**	**36**	100.0	100.0	100.0	97.2	91.7	77.8	61.1	44.4	38.9	13.9
**DvCA_Single**	**84**	100.0	100.0	100.0	97.6	92.9	77.4	57.1	39.3	33.3	13.1
***Aci*SHC_Final**	**4**	100.0	75.0	75.0	75.0	75.0	75.0	75.0	50.0	50.0	50.0
***Aci*SHC_Single**	**9**	100.0	100.0	88.9	88.9	77.8	77.8	55.6	44.4	44.4	11.1
**Average**		100.0	95.8	93.3	91.8	87.6	77.2	62.5	48.8	44.6	28.6

Table S1: Extended overview of different evolution campaigns and how improved variants are distributed concerning ΔΔG, calculated through the rosetta cartesian ΔΔG protocol. This table includes the additional entries DvCA_Single and *Aci*SHC_Single. DvCA_Single refers to the 84 single-point mutations obtained during the NNK screening (as described in the main text). The *Aci*SHC_Single row refers to the nine single-point mutations initially identified as beneficial of which only four were included in the final SHC variant.

Sequence space (%)

	# mut	100.0	90	80	70	60	50	40	30	20	10
**ATA217**	**26**	100.0	92.3	80.8	65.4	57.7	57.7	46.2	42.3	42.3	30.8
**HG3.17**	**17**	100.0	94.1	94.1	82.4	76.5	58.8	35.3	29.4	23.5	11.8
**DvCA_Final**	**36**	100.0	100.0	97.2	94.4	91.7	75.0	61.1	55.6	38.9	19.4
**DvCA_Single**	**84**	100.0	100.0	96.4	91.7	91.7	82.1	67.9	58.3	41.7	21.4
***Aci*SHC_Final**	**4**	100.0	75.0	75.0	50.0	50.0	25.0	25.0	0.0	0.0	0.0
***Aci*SHC_Single**	**9**	100.0	66.7	66.7	66.7	66.7	66.7	66.7	33.3	22.2	11.1
**average**		100.0	88.0	85.0	75.1	72.4	60.9	50.3	36.5	28.1	15.8

Table S2: Extended overview of different evolution campaigns and how improved variants are distributed concerning ΔΔG, calculated with the ACDC-NN model. This table includes the additional entries DvCA_Single and *Aci*SHC_Single. DvCA_Single refers to the 84 single-point mutations obtained during the NNK screening (as described in the main text). The *Aci*SHC_Single row refers to the nine single-point mutations initially identified as beneficial of which only four were included in the final SHC variant.

Sequences:

> sequence A

TAATACGACTCACTATAGGGATGGCAGAAGCAGCACAGAGCGTTGACCAGCAAATTAAAGCACGTGGTAAAGTT
TATTTTGGTGTTGCCACCGATCAGAATCGTCTGACCACCGGTAAAAATGCAGCAATTATTCAGGCAGATTTTGGTA
TGGTTTGGCCTGAAAATAGCATGAACTGAGCAATAACTAGCATAA

> sequence B

TAATACGACTCACTATAGGGATGGCAGAAGCAGCACAGAGCGTTGACCAGCTGATTAAAGCACG
TGGTAAAGTTTATTTTGGAAATGCCACCGATCAGAATCGTCTGACCACCGGTAAAAATGCAGCAA
TTATTCAGGCAGATTTTGGTATGGTTTGGCCTGAAAATAGCATGAACTGAGCAATAACTAGCATAA

> sequence C

TAATACGACTCACTATAGGGATGGCAGAAGCAGCACAGAGCGTTGACCAGCTGATTAAAGCACG
TGGTAAAGTTTATTTTGGTGTTGCCACCGATCAGAATCGTCTGACCACCAAAAAAAATGCAGCAA
TTATTCAGGCAGATTTTGGTATGGTTTGGCCTGAAAATAGCATGAACTGAGCAATAACTAGCATAA

> sequence D

TAATACGACTCACTATAGGGATGGCAGAAGCAGCACAGAGCGTTGACCAGCTGATTAAAGCACGTGGTAAAGTT
TATTTTGGTGTTGCCACCGATCAGAATCGTCTGACCACCGGTAAAAATGCAGCAATTATTCAGGCAGATTAAAGT
ATGGTTTGGCCTGAAAATAGCATGAACTGAGCAATAACTAGCATAA

References

[1] Novick SJ, Dellas N, Garcia R, Ching C, Bautista A, Homan D, et al. Engineering an amine transaminase for the efficient production of a chiral sacubitril precursor. ACS Catal 2021;11:3762–70. https://doi.org/10.1021/acscatal.0c05450.

[2] Alvizo O, Nguyen LJ, Savile CK, Bresson JA, Lakhapatri SL, Solis EOP, et al. Directed evolution of an ultrastable carbonic anhydrase for highly efficient carbon capture from flue gas. Proc Natl Acad Sci U. S. A. 2014;111:16436–41. https://doi.org/10.1073/pnas.1411461111.

[3] Blomberg R, Kries H, Pinkas DM, Mittl PRE, Grütter MG, Privett HK, et al. Precision is essential for efficient catalysis in an evolved Kemp eliminase. Nature 2013;503:418–21. https://doi.org/10.1038/nature12623.

[4] Eichenberger M, Hüppi S, Patsch D, Aeberli N, Berweger R, Dossenbach S, et al. Asymmetric Cation-Olefin Monocyclization by Engineered Squalene–Hopene Cyclases. Angew Chem Int Ed 2021;60:26080–6. https://doi.org/10.1002/anie.202108037.
